# Supplementary material for: Anomalous behavior of critical current in a superconducting film triggered by DC plus terahertz current
Source: Nat Commun. 2024 May 24;15:4435. doi: 10.1038/s41467-024-48738-8 (PMC11126563; doi:10.1038/s41467-024-48738-8)
Supplement: Supplementary file 1 — Supplementary Information [file 41467_2024_48738_MOESM1_ESM.pdf]

Supplemental Information for

Anomalous behavior of critical current in a superconducting film triggered by DC plus terahertz current

Fumiya Sekiguchi*, Hideki Narita, Hideki Hirori, Teruo Ono and Yoshihiko Kanemitsu*

Institute for Chemical Research, Kyoto University, Uji, Kyoto 611-0011, Japan
*Correspondence to: sekiguchi@crc.u-tokyo.ac.jp, kanemitu@scl.kyoto-u.ac.jp

S1. Methods

Figure S1-1**a** shows a schematic picture of the experimental setup. Artificial noncentrosymmetric superlattices made of stacked SC elements were demonstrated to show a clear SC diode effect (SDE) under the external magnetic field or the adjacent magnetic elements, as a consequence of broken spatial and time reversal symmetry[1,2]. A distinctive feature of the artificial superlattices is that, owing to the steepness of their SC transitions, they can show binary (very-high-rectification-ratio) SC/normal state switching by alternating the polarity of the current or the magnetic field. Among the elements in the periodic table, we focused on Nb, V, and Ta, which have high T_c and close lattice constants. When these three elements are stacked in this order, a polar axis in the stacking direction emerges which breaks the spatial inversion symmetry. The thickness of each layer (2 nm) and the total thickness (30 nm) were determined so that the superlattice was thin enough while it had high enough T_c (here, 4 K). The former allows us to perform THz transmission spectroscopy (thick metal films do not allow THz pulses to transmit with a measurable amplitude), while the latter leads to a relatively large SC gap energy of few meV, which can be observed in the THz spectral window.

The artificial superlattice [Nb (2.0 nm)/V (2.0 nm)/ Ta (2.0 nm)]$_5$ was epitaxially grown on MgO (100) substrate at 700 °C by direct current magnetron sputtering in a high-vacuum system with a base pressure of approximately 5×10^{-6} Pa. DC currents were injected into the superlattice through Ti/Au metal electrodes deposited on both ends of the film sample, as shown in Fig. S1-1. The DC current injection and the two-terminal current-voltage (*I*-*V*) measurement were performed by using a DC voltage current source/monitor. The sample was cooled down in a magneto-optical cryogen-free superconducting magnet system. The magnetic field was applied in the plane of the film and perpendicular to the DC current.

For the THz spectroscopy, we used a wide-area film sample (4-mm square) because of the large spot size of THz pulses. On the other hand, for the detailed investigation of the critical current I_c', another film sample was patterned into a 50-μm-wide and 900-μm-long wire structure by using a conventional photolithography and Ar ion milling process. The optical images of the samples are shown in Fig. S1-1**b,c**.

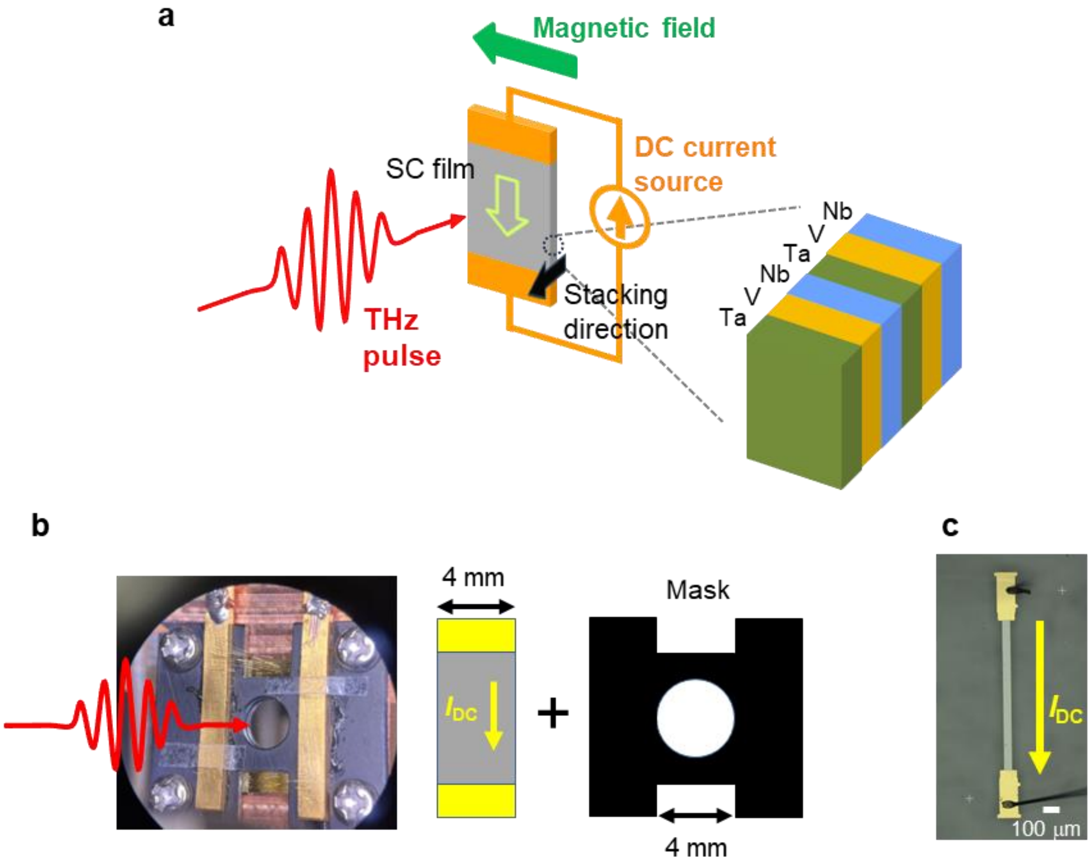

Fig. S1-1. a Illustration of the experimental setup. E_{THz} was either parallel or perpendicular to the DC current. **b** Optical image and schematic drawing of the wide-area sample for THz spectroscopy. **c** Optical image of the sample for the detailed measurement of critical currents, which was fabricated into a wire structure.

An illustration of the setup for THz pulse generation and transmission spectroscopy is shown in Fig. S1-2. For a fundamental light source, we used a Ti:Sapphire regenerative amplifier system with a center photon energy of 1.55 eV, pulse duration of 80 fs, and a repetition rate of 1 kHz. THz pulses were generated by optical rectification in a $LiNbO_3$ crystal using the tilted-pulse-intensity-front scheme. Before irradiating the sample, the electric field strength of the THz pulses ($E_{\rm THz}$) was changed by a pair of wire-grid polarizers. For the experiment of narrowband excitations, the THz pulses were spectrally narrowed by metal mesh band-pass filters (from Origin Ltd). $E_{\rm THz}$ of the THz pulses was either parallel or perpendicular to the DC current. THz spectroscopy was performed in the transmission geometry.

The $E_{\rm THz}$ strength of detected THz pulses was estimated by the EO sampling in a (110) ZnTe crystal with the thickness of l = 200 μm, using the relation

$$E_{\rm THz} = \frac{\lambda}{2\pi n_0^3 l r_{41} t_{\rm THz}} \frac{\Delta I}{I}$$

where n_0 = 2.8 is the refractive index of ZnTe at the wavelength λ = 800 nm of the gate pulse, r_{41} = 4 pm/V is the EO coefficient, $t_{\rm THz} = 2/(n_{\rm THz} + 1)$ with $n_{\rm THz}$ = 3.17 is the Fresnel transmission coefficient at the surface of ZnTe crystal. $\Delta I/I$ is the normalized signal amplitude from the balanced photodiode. The $E_{\rm THz}$ strength at the sample position was estimated to be 1/4 of that measured at the EO sampling position, taking into account the larger spot size of THz pulses at the sample position, by a factor of 4, compared to the spot size at the EO sampling position.

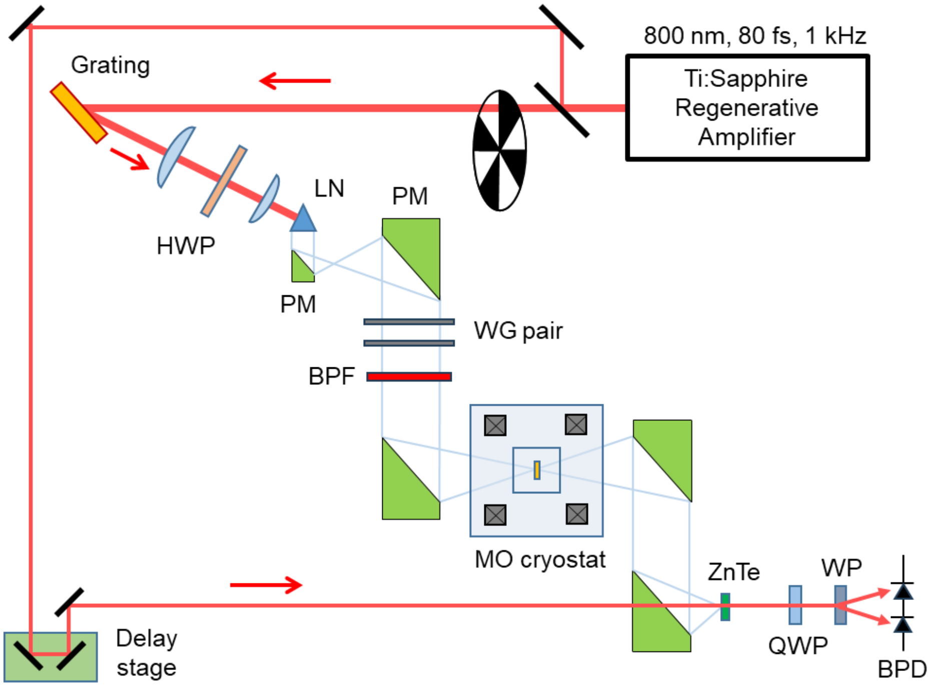

Fig. S1-2. Experimental setup for THz pulse generation and spectroscopy. LN: $LiNbO_3$ crystal, HWP: half-wave plate, PM: parabolic mirror, WG: wire grid polarizer, BPF: band-pass filter, MO cryostat: magneto-optical cryostat, QWP: quarter-wave plate, WP: Wollaston prism, BPD: balanced photodiode.

S2. Temperature dependence of the critical magnetic field, with and without THz pulse irradiation

To check whether the temperature of the sample was increased due to the THz irradiations, we measured the critical magnetic field. Figure S2 shows the resistance as a function of the applied in-plane magnetic field, measured at different temperatures. When the external magnetic field reaches the critical magnetic field, the resistance jumps to a high value. Figure S2 shows that the critical magnetic field sensitively changes depending on the sample temperature. On the other hand, the THz irradiation hardly affects the critical magnetic field. This insensitivity to the THz irradiation confirms that the temperature increase due to the THz excitation is negligible.

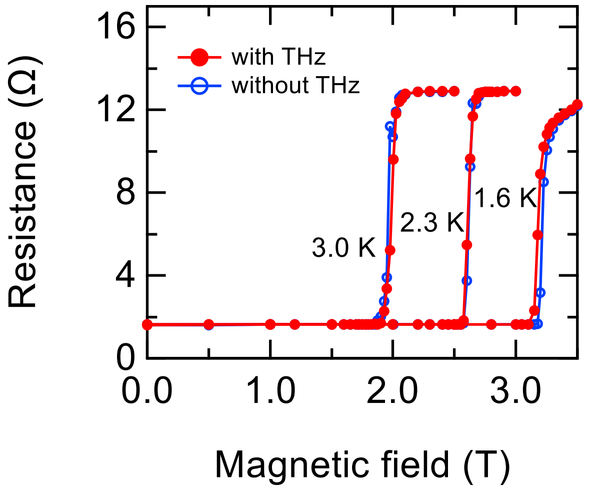

Fig. S2. Resistance as a function of the magnetic field, measured at different temperatures. Red (blue) points show sample data with (without) the THz irradiation.

S3. Complex THz conductivity spectra

For a complete dataset, here we show the complex THz conductivity spectra, including the imaginary part. Fig. S3**a** compares the complex conductivity spectra at 8.0 K and 1.6 K, measured at zero DC current and zero magnetic field. Whereas the spectrum at 8.0 K is featureless, the real part of the conductivity is suppressed at 1.6 K in the low-energy region, indicating the formation of the SC gap. The transition to the SC state can be also identified in the imaginary part of the conductivity at 1.6 K, which shows the $1/\omega$-type feature below 2 meV.

As explained in the main text, the THz conductivity spectrum hardly changes depending on the DC current amplitude, both in the real and imaginary part, as shown in Fig. S3**b**.

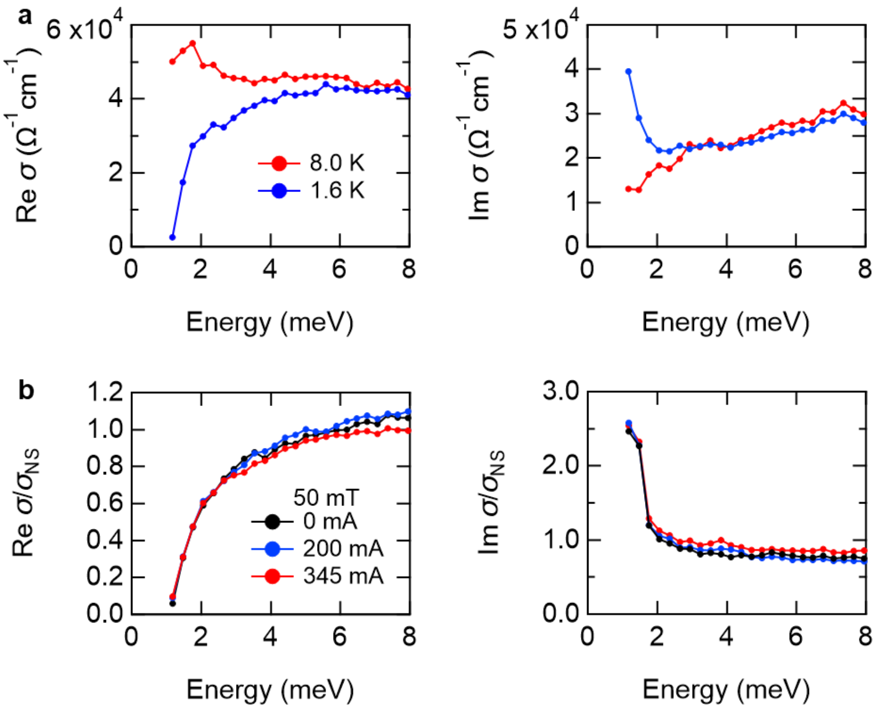

Fig. S3. a Real (left) and imaginary (right) part of the THz conductivity spectra, at 8.0 K and 1.6 K, without the DC current and external magnetic field. **b** Real (left) and imaginary (right) part of the THz conductivity spectra, measured at 1.6 K, 50 mT, and different I_{DC}.

S4. Magnetic field dependence of the THz conductivity spectra and SC gap energy

Here we discuss how the in-plane magnetic field affects the SC gap energy Δ. Figure S4**a** shows the in-plane magnetic field dependence of the THz conductivity spectra, measured without applying the DC current. The temperature was 1.6 K, and the spectra were normalized by the normal-state THz conductivity measured at 8.0 K. With increasing magnetic field, the low-energy spectral weight in the real part continuously recovers, and the $1/\omega$ component in the imaginary part disappears. These features indicate that the SC gap is continuously suppressed by the magnetic field. The spectra can be fitted with the Mattis-Bardeen model for dirty-limit SCs, allowing the SC gap energy Δ to be extracted. Δ is plotted in Fig. S4**b** as a function of the magnetic field. While the SC gap continuously shrinks to become zero at a critical field around 3.2 T, the gap energy remains almost intact in the low magnetic field region below 100 mT, where the SDE appears.

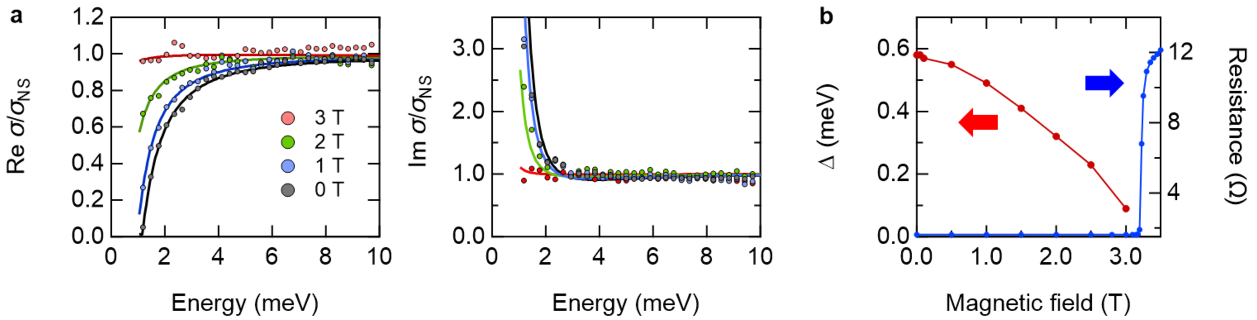

Fig. S4. a THz conductivity spectra at different in-plane magnetic fields. The temperature is 1.6 K, and the spectra are normalized by the normal-state THz conductivity spectrum at 8.0 K. Solid lines are fits by the Mattis-Bardeen model. **b** In-plane magnetic field dependence of the SC gap energy Δ obtained from the fits shown in S4**a**. The simultaneously measured resistance shows that the critical magnetic field is 3.2 T.

S5. Nonlinearity induced solely by the THz pulse

Here we discuss the nonlinear effect induced solely by the broadband THz pulse. Figure S5**a** shows the E_{THz}-dependence of the real-part THz conductivity spectra, measured without DC currents. At low E_{THz}, the SC gap is clearly discerned. As E_{THz} increases above 14 kV/cm, the SC gap continuously shrinks, leading to the recovery of the spectral weight below 4 meV. Such a gap closing by intense-THz-pulse excitations can be attributed to the impulsive photoinjection of quasiparticles[3]. In particular, the broadband THz pulses used for the THz spectroscopy contain photons whose energies are higher than the SC gap, which are directly absorbed to form quasiparticles.

We show the E_{THz} dependence of the spectral weight around the SC gap in Fig. S5**b**. Here, the magnitude of "lost" spectral weight below 4 meV, as indicated by the shaded area in Fig. S5**a**, is plotted so that it can be directly related to the SC gap energy. Figure S5**b** shows that the SC gap continuously shrinks with increasing E_{THz}, without any threshold. Therefore, the nonlinearity originating solely from the THz pulses grows continuously.

In Fig. S5**b**, we also plot the simultaneously measured critical current I_c' depending on the E_{THz} strength. This corresponds to the data shown in Fig. 3 in the main text, but measured with the broadband THz pulse. Here, a significant reduction in I_c' occurs already at the lowest E_{THz} of 4.6 kV/cm, which is almost the weakest E_{THz} that allows transmission spectroscopy with satisfactory SNR. Then, as E_{THz} increases, I_c' stays around 300 mA without a significant reduction. This behavior clearly differs from the nonlinearity triggered by the THz pulse only. Therefore, to explain the sensitive reduction in the critical current triggered by the THz pulse excitation, we need to consider the "interplay" between THz pulses and DC current.

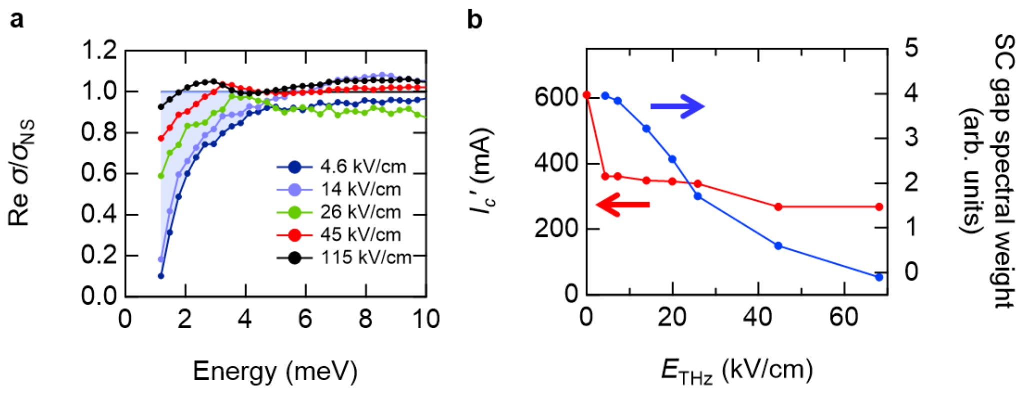

Fig. S5. a E_{THz} dependence of the real part of THz conductivity spectra, normalized by the normal-state THz conductivity measured at 8.0 K. **b** E_{THz} dependence of I_c' and the lost spectral weight around the gap energy, as shown by the shaded area in S5**a**.

S6. Spectra and waveforms of the THz pulses

As shown in Fig. S1-2, the narrowband THz pulses were obtained by using THz band-pass filters inserted in the optical path of the relatively broadband THz pulse, which was generated from a $LiNbO_3$ crystal. The waveform and spectrum of the input broadband THz pulse are shown in Fig. S6**b**. The spectra of narrowband THz pulses with different frequencies are shown in Fig. S6**a**, where they are plotted with the real part of the SC conductivity spectrum at 1.6 K. The 2.0-THz and 0.8-THz pulses have energies higher than 2Δ, enabling them to induce quasiparticle excitations. On the other hand, the 0.2-THz and 0.3-THz pulses have energies smaller than or similar to 2Δ, which can be seen in Fig. S6**a** where the real part of the THz conductivity has tiny weights in the spectral region of those lower-frequency pulses. This indicates that the amount of THz-induced diffusive current is tiny for the lower-frequency excitations. Figure S6**a** also shows that the spectral width of the THz pulses becomes larger for higher-frequency THz pulses. Indeed, the time duration of the THz pulses becomes shorter for higher frequencies, as shown in Fig. S6**c**. The time durations of the 0.2-, 0.3-, 0.8- and 2.0-THz pulses were 39 ps, 23 ps, 4.4 ps and 2.5 ps, respectively, obtained by Gaussian fits to the pulse envelopes.

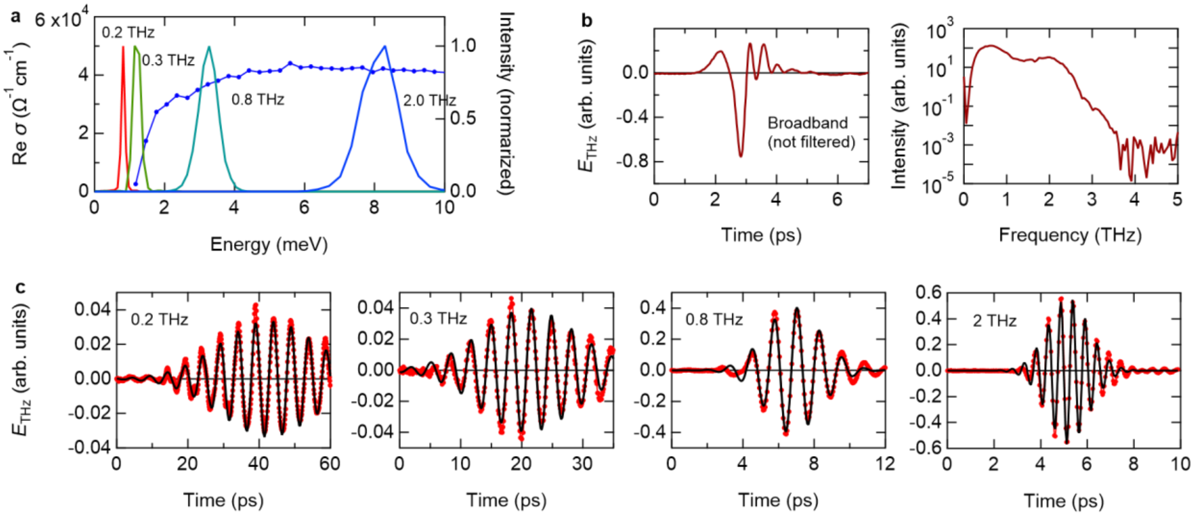

Fig. S6. a Spectra of the 0.2-, 0.3-, 0.8- and 2.0-THz pulses. The line with blue circles shows the real part of the conductivity spectrum of the SC state at 1.6 K. **b** Waveform and spectrum of the input broadband THz pulse generated from a $LiNbO_3$ crystal. **c** E_{THz} waveforms of the 0.2-, 0.3-, 0.8- and 2.0-THz pulses. Solid lines are the fits assuming a Gaussian envelope and sinusoidal oscillation.

S7. THz-induced current amplitude in the SC state

The current amplitude in the film sample induced by the THz pulses was calculated by

$$I_{\mathrm{THz}}(\omega) = \sigma(\omega) E'_{\mathrm{THz}}(\omega) d_{\mathrm{film}} w$$

where $\sigma(\omega)$ is the optical conductivity of the SC state, which is shown in Fig. S7. $d_{\mathrm{film}} w$ is the cross-sectional area of the film sample, where d_{film} = 30 nm is the thickness and w = 50 μm is the width. $E'_{\mathrm{THz}}(\omega)$ is the THz electric field inside the sample, calculated taking into account the multiple reflection inside the thin film, represented as

$$E'_{\mathrm{THz}}(\omega) = t\left(\frac{1}{1 - r_1 r_2 e^{2i\omega n(\omega) d_{\mathrm{film}}/c}} + \frac{r_1 e^{2i\omega n(\omega) d_{\mathrm{film}}/c}}{1 - r_1 r_2 e^{2i\omega n(\omega) d_{\mathrm{film}}/c}}\right) E_{\mathrm{THz}}(\omega)$$

where $n(\omega)$ is the complex refractive index, $t = 2/(1 + n(\omega))$ and $r_1 = (n_{\mathrm{MgO}} - n(\omega))/(n_{\mathrm{MgO}} + n(\omega))$, $r_2 = (1 - n(\omega))/(1 + n(\omega))$ are the transmission and reflection coefficients.

Figure S7 shows the spectral overlap of the 0.3 THz excitation pulse with the complex THz conductivity of the SC state at 1.6 K. Using the above equations, the 0.3-THz pulse with E_{THz} = 1 kV/cm is calculated to induce a current I_{THz} = 24 mA flowing in the film sample. Therefore, the magnitude of I_{THz} is comparable to or larger than the I_c. These values were taken into account in the model for THz-current-assisted vortex depinning.

We note that, in the vortex depinning model, the THz current alone does not induce a persistent SC breakdown even if the I_{THz} amplitude is larger than I_c. This is because the THz-induced oscillating current ceases within a short duration of ps scale. A long-lasting motion of the vortex cannot be induced without a DC field, and hence, the vortex relaxes back to the trap potential after the THz oscillation decays.

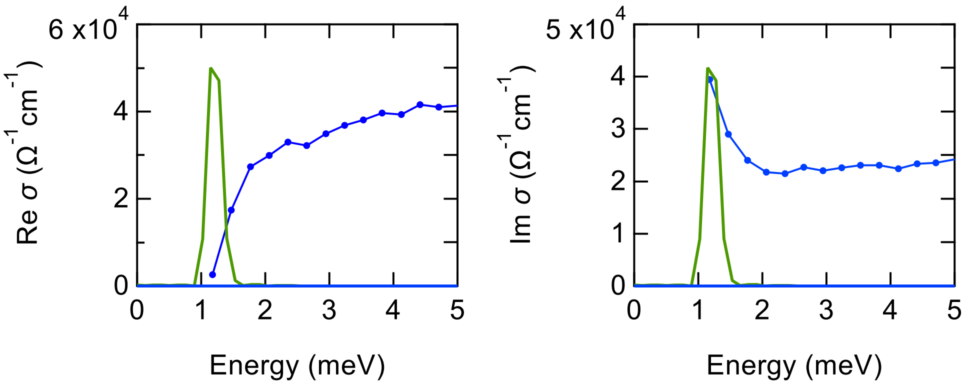

Fig. S7. Real and imaginary part of the THz conductivity at 1.6 K, plotted with the spectrum of the 0.3-THz pulse.

S8. Comparison of the E_{THz}-dependence of the critical current at 0.2-THz and 0.3-THz excitations

In the experiment, the narrowband THz pulses were obtained by using THz band-pass filters inserted in the optical path of the relatively broadband THz pulse, which was generated from a $LiNbO_3$ crystal. Therefore, the maximum E_{THz} strength available was determined by the power spectrum of the initial broadband THz pulse, and hence, was different depending on the frequency. This is the reason why the scale of horizontal axes in Fig. 3 in the main text differs depending on the excitation frequency.

It would be useful to compare the behavior of the critical current I_c' at 0.2-THz and 0.3-THz excitations, which have comparable or smaller phonon energy than the optical gap 2Δ. In Fig. S8, we plot the E_{THz}-dependence of I_c' at 0.2-THz and 0.3-THz excitations on the same horizontal scale. The result shows that the behavior at 0.3 THz and 0.2 THz are similar. The difference between the two can be attributed to the different SC conductivity at 0.2 THz and 0.3 THz, and also to the different pulse widths of the 0.2-THz and 0.3-THz pulse.

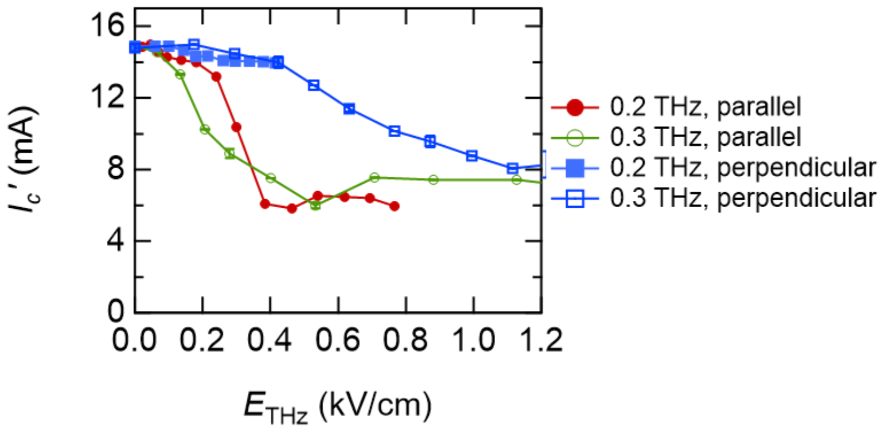

Fig. S8. E_{THz} dependence of the critical current I_c' at the excitation of 0.2 THz and 0.3 THz (zoomed-in view of Fig. 3**c**&**d** in the main text).

S9. Errors in the I_c' measurements

In the data set shown in Fig. 3 in the main text, we repeated the scan several times at 0.3-THz excitation, and we estimated the errors by evaluating standard deviations. In addition, we also show the results of two scans at the 0.8-THz excitation, measured with two different sets of E_{THz} values, in Fig. S9. The comparison of the two scans provides an estimation of the precision of the I_c' measurement.

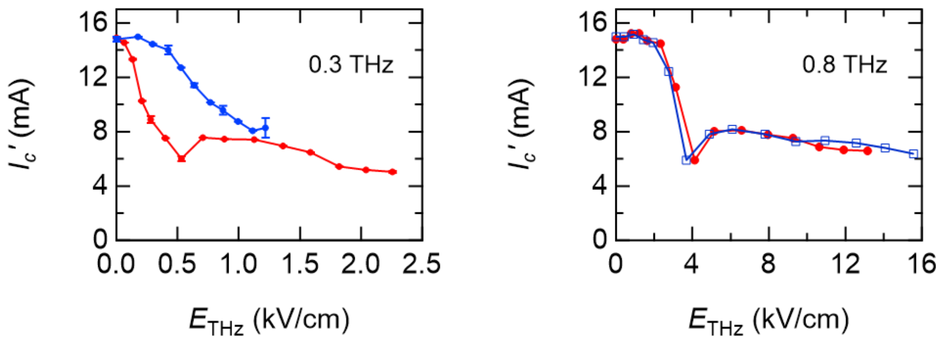

Fig. S9. E_{THz} dependence of the critical current I_c' at the excitation of 0.3 THz and 0.8 THz. The plot for 0.3 THz includes error bars, and the plot for 0.8 THz shows the results of two scans with different sets of E_{THz} values.

S10. Pulsed DC currents

For the measurement shown in Fig. 4 in the main text, we used pulsed DC currents to minimize the Joule heating. While the use of pulsed DC currents increases noises in the voltage measurements, it allows us to perform the *I*-*V* measurement in the I_{DC} region above I_c'. This is owing to the small duty ratio, a 200-μs-duration DC current pulse flows every 20 ms, resulting in a small amount of Joule heating. The low repetition rate also allows the sample to relax back to the initial state before the next pulse event occurs.

Taking into account the shortest pulse duration for the *I*-*V* measurement available with our experimental setup, and also the μs-scale time jitter between the DC current and THz pulses (they were synchronized), we chose to use 200-μs-duration isolated square pulses for the DC current. A THz pulse irradiated the sample 50 μs after the DC current ramped up, as schematically shown in Fig.S10. Concerning the repetition rate, the lower is better in terms of Joule heating, but it sacrifices SNR. In this work, we chose 50 Hz.

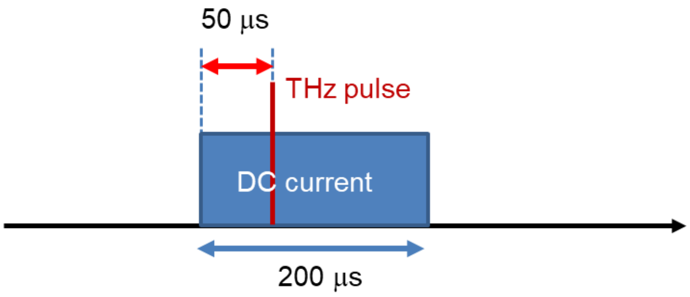

Fig. S10. Schematic picture of the synchronized DC current and THz pulse.

S11. Direction dependence of the critical magnetic field

Figure S11 compares the temperature dependence of the critical magnetic field when the field is applied parallel or perpendicular to the film plane. The critical magnetic field is defined as the field value at which the resistance jumps, as can be seen in Fig. S2. Figure S11 shows that the out-of-plane magnetic field much more efficiently suppresses the SC state than the in-plane magnetic field does.

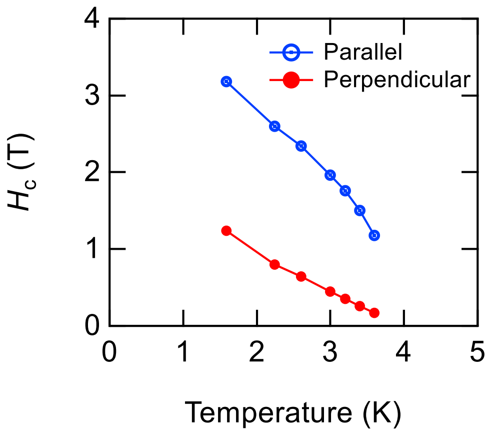

Fig. S11. Temperature dependence of the critical magnetic field when the field is applied parallel or perpendicular to the film plane.

S12. THz-pulse-duration dependence of the critical current

As shown in Fig. S6, the time duration of the THz pulses differs depending on the filtered frequency. Here, to assess the effect of the pulse-duration difference on the critical current reduction, we calculate I_c' using I_{THz} with different pulse durations. As shown in Fig. S12, I_c' behaves almost the same for durations of 23 ps and 10 ps. Upon further reduction in the pulse duration, I_c' becomes less sensitive to I_{THz}, while it still decreases monotonically at the shortest duration of 2.5 ps. Therefore, though the duration of the THz pulses does affect the I_c' modification, its impact is modest compared to that comes from the difference in frequency, as shown in Fig. 5**d** in the main text.

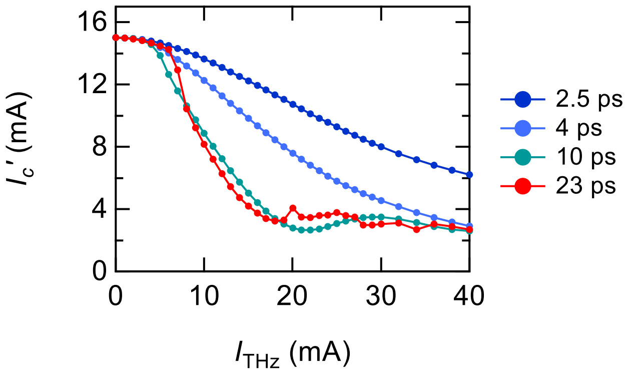

Fig. S12. I_{THz} dependence of I_c' calculated for THz pulses with different time durations. THz frequency is 0.3 THz.

S13. *I-V* curves under THz excitations with different E_{THz} strengths

As shown in Fig. 4 in the main text, a non-monotonic SC/normal state transition appears in the *I-V* curve under the 0.3-THz excitation with E_{THz} = 0.9 kV/cm. On the other hand, the *I-V* curve shows a monotonic behavior with a single critical current at a higher E_{THz} of 2.3 kV/cm, as shown in Fig. S13**b**. The sensitivity to the E_{THz} strength can also be seen in the simulated results in Fig. S13**c**. At I_{THz} = 8 mA, a wide region of vortex repinning appears above I_c', as discussed in the main text. However, at higher excitations of I_{THz} = 20 mA and 40 mA, the repinning regions above I_c' are very narrow. Such a spike-like vortex repinning can be obscured by the inhomogeneity of the trap potential or the fluctuation of the THz excitation strength. Hence, although the vortex repinning is, in theory, possible at stronger THz excitations, it was not observed in the experiment. This is consistent with the experimental result in Fig. S13**a,b**.

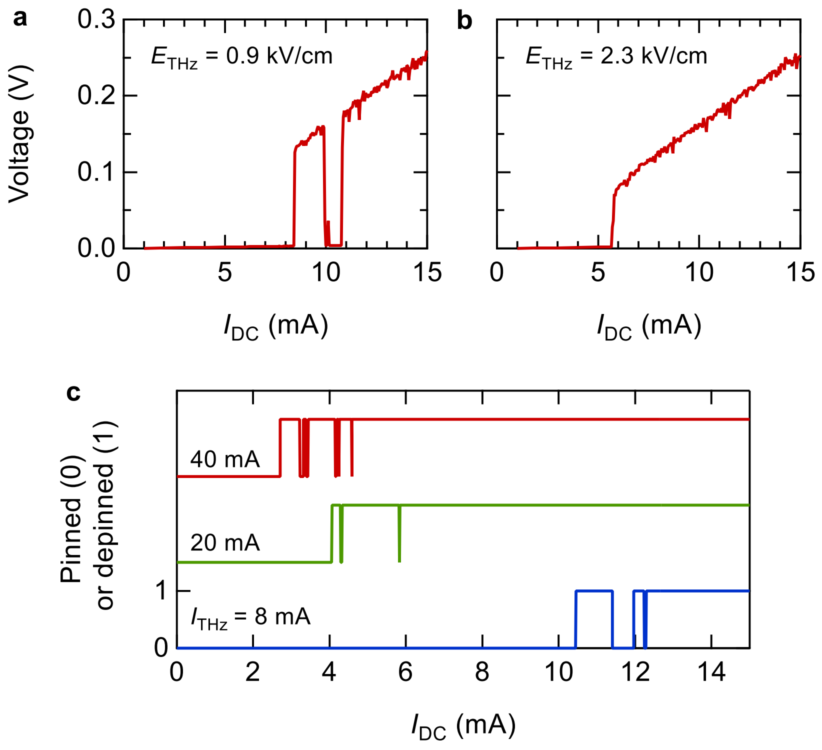

Fig. S13. *I-V* curve measured under THz excitations of strength **a** E_{THz} = 0.9 kV/cm and **b** E_{THz} = 2.3 kV/cm. The temperature is 1.6 K and the frequency is 0.3 THz. **c** Simulated I_{DC} dependence of the vortex depinning for different peak amplitudes of I_{THz}.

S14. Vortex repinning at different THz current amplitudes and frequencies

Here we show how the simulated vortex repinning region depends on I_{THz} and its frequency. As shown in Fig. S14**a**, I_r indicates the DC current where the vortex repinning first occurs, and we plot the simulated I_{THz} dependence of I_c', I_r and I_c'' at 0.4-THz, 0.3-THz and 0.2-THz excitations in Fig. S14**b**. The width of the I_{DC} region where the vortex repinning occurs, I_c''− I_r, is plotted in Fig. S14**c**.

While the results are quantitatively different depending on the THz frequency, a common trend can be seen in the repinning behavior: the vortex repinning occurs in a wide I_{DC} region when excited by an intermediate I_{THz} strength. Figure S14**b** shows that, as I_{THz} increases, I_c' starts to decrease drastically at a certain I_{THz} (around 8 mA for 0.3 THz, for example) before it saturates at a low value (~ 4 mA). As shown in Fig. S14**c**, a sizable repinning region appears at this intermediate region. This simulated behavior is consistent with the experimental result. We note that the critical current becomes less sensitive to I_{THz} at higher frequencies, as shown in Fig. 5**d** in the main text. In this case, the THz current does not assist vortex depinning, and hence, vortex repinning also does not occur.

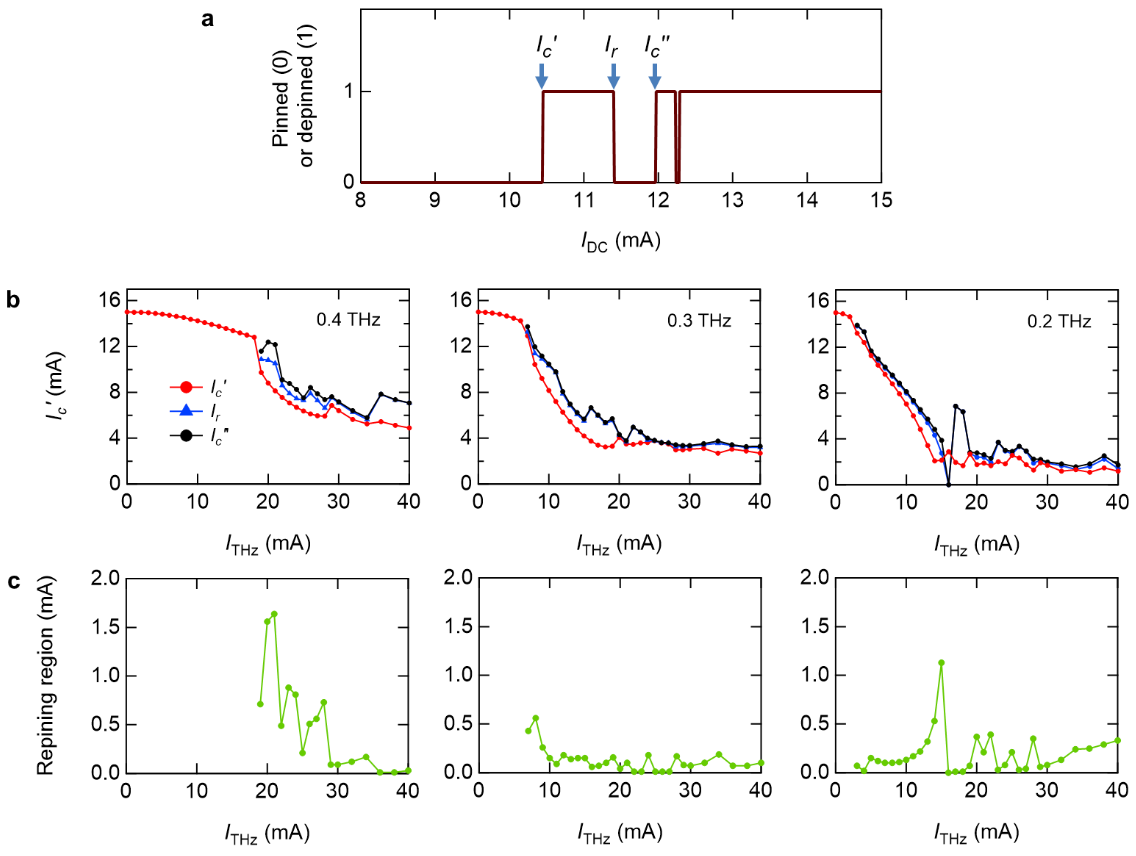

Fig. S14. a Plot indicating whether the vortex is pinned or depinned depending on I_{DC}, under the influence of I_{THz}. I_r indicates the value of I_{DC} where the repinning first occurs. **b** I_{THz} dependence of I_c', I_r and I_c'' at the excitation of 0.4 THz, 0.3 THz and 0.2 THz. **c** I_{THz} dependence of the width of the vortex repinning region, i.e., I_c''− I_r .

S15. THz-current-induced vortex motion at different DC currents

Figure S15 shows complementary plots for Fig. 5**a,b** and Fig. 6**a** in the main text; the trap potential and the vortex motion calculated at different I_{DC}. The THz excitation frequency is 0.3 THz and the peak amplitude is I_{THz} = 8 mA. Figure S15 clearly shows that vortex depinning occurs non-monotonically with increasing I_{DC}, while the minimum of the trap potential becomes continuously shallower.

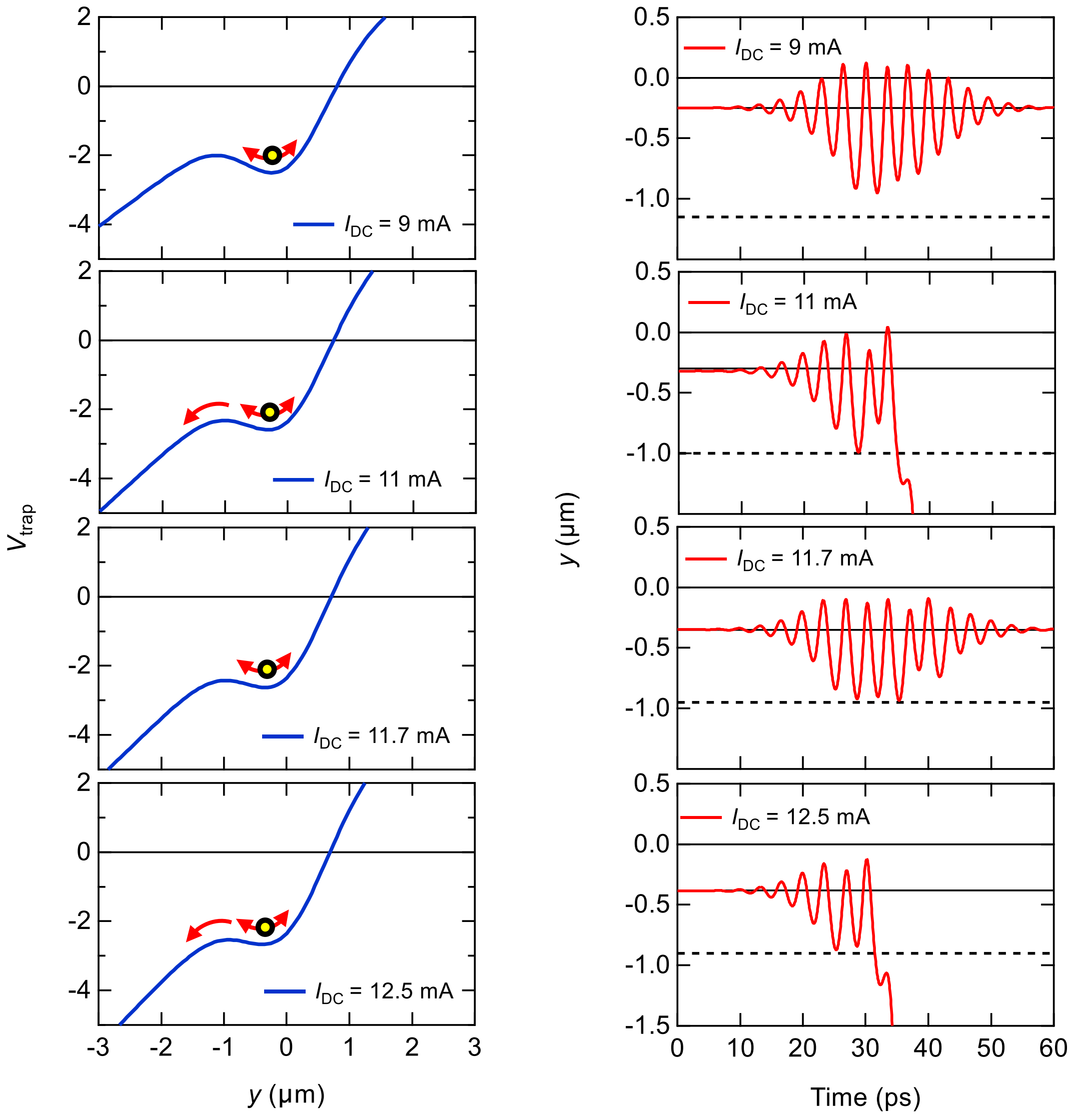

Fig. S15. Trap potential and vortex motion calculated at different I_{DC}. The frequency is 0.3 THz and I_{THz} = 8 mA.

S16. Nonreciprocity of critical current with the THz excitations

As mentioned in the main text, our artificial superlattice shows the SDE, i.e., nonreciprocity of the critical current ΔI_c', without the THz irradiation. Here, we discuss how the SDE is affected by the THz excitation. Figure S16**a,c** plot the measured values of I_c' and ΔI_c', respectively, as a function of E_{THz} strength. The temperature is 1.6 K, and the in-plane magnetic field of 50 mT is applied perpendicular to the DC current. These experimental results indicate that the magnitude of ΔI_c' is suppressed as soon as I_c' is reduced by the THz excitation.

Next, we simulated the situation by using a modified model of THz-assisted vortex depinning. To reproduce the nonreciprocal critical current at I_{THZ} = 0 mA, we introduced an asymmetry in the trap potential,

$$V_{\mathrm{trap}} = - - d_{\mathrm{film}} \Phi_0 J_{\mathrm{C}} \sqrt{\frac{e}{\alpha}} \left(1 + \frac{y}{A}\right) \exp\left[-\frac{\alpha}{2} y^2\right]$$

where A is the parameter that induces the distortion in the potential. We used a value of A that gives a finite nonreciprocity ΔI_c' at I_{THZ} = 0 mA while reproducing the experimental result of E_{THz} dependence of I_c'. The calculated results are shown in Fig. S16**b,d**. Here, ΔI_c' is severely affected in the region where I_c' is modified by I_{THz}. The modified value of ΔI_c' can be tiny or have opposite signs depending on the I_{THz}, and hence, the original nonreciprocity is obscured. This can be understood considering that the depinning of the vortex is dominated by the I_{THz}-induced vortex oscillation with a large amplitude, where the delicate asymmetric structure of the trap potential does not play a decisive role.

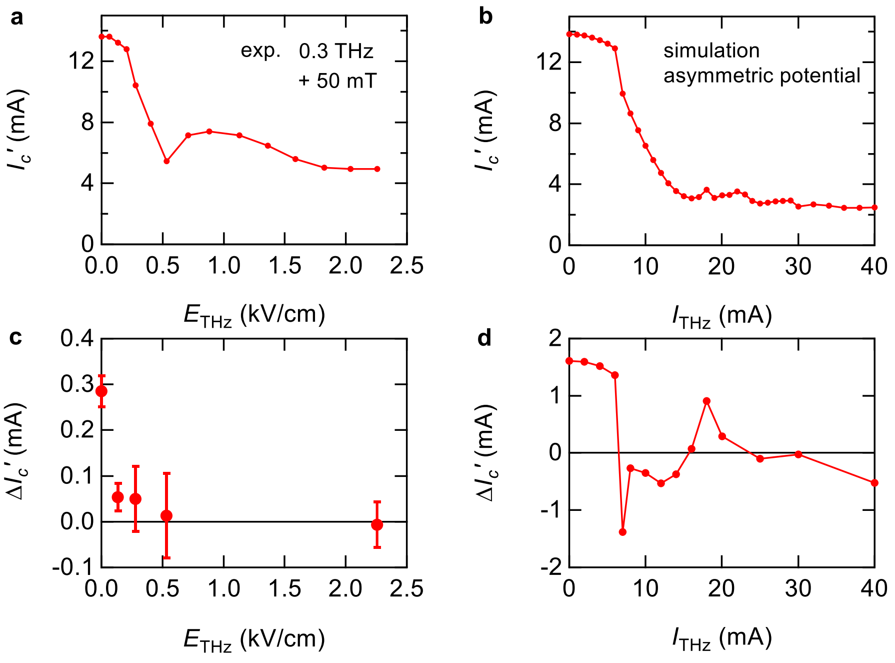

Fig. S16. a Measured E_{THz} dependence of I_c' under THz excitation of 0.3 THz and in-plane magnetic field of 50 mT. **b** Simulated I_{THz} dependence of I_c' assuming an asymmetric trap potential. **c** Measured nonreciprocity of I_c' as a function of E_{THz}. **d** Simulated nonreciprocity of I_c' as a function of I_{THz}.

S17. Attribution of the low resistive state emergent above I_c'

As shown in Fig. 4 in the main text, a low resistive state appears at I_{DC} above I_c' under the specific THz excitation. Here, it is not trivial whether the re-entrant-like feature reflects the formation of a true SC state or some non-equilibrium state of highly mobile carriers. We do not exclude the possibility of the latter case, while we do not have an experimental result to support it. On the other hand, it is reasonable to assume the former case because the resistivity of the sample becomes almost zero (not “simply low”). This can be confirmed in Fig. S17, which shows the zoomed-in view of Fig. 4 in the main text. Note that the finite gradient of the *I*-*V* curve in the SC state comes from the resistance of the lead wires.

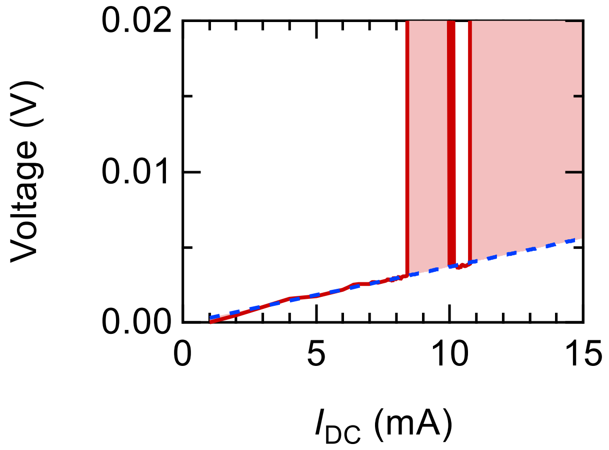

Fig. S17. Zoomed-in view of Fig. 4 in the main text; *I*-*V* curve under the THz excitation. THz frequency is 0.3 THz and E_{THz} = 0.9 kV/cm. The blue dashed line shows the finite gradient of the *I*-*V* curve in the SC state, originating from the resistance of the lead wires.

Supplementary References

1. F. Ando, Y. Miyasaka, T. Li, J. Ishizuka, T. Arakawa, Y. Shiota, T. Moriyama, Y. Yanase, and T. Ono, *Observation of Superconducting Diode Effect*, Nature **584**, 373 (2020).
2. H. Narita et al., *Field-Free Superconducting Diode Effect in Noncentrosymmetric Superconductor/Ferromagnet Multilayers*, Nat. Nanotechnol. **17**, 823 (2022).
3. R. Matsunaga and R. Shimano, *Nonequilibrium BCS State Dynamics Induced by Intense Terahertz Pulses in a Superconducting NbN Film*, Phys. Rev. Lett. **109**, 187002 (2012).
